# Surgical outcomes and their relation to the number of prior episodes of diverticulitis

**DOI:** 10.1093/gastro/got017

**Published:** 2013-07-09

**Authors:** Shota Takano, Cesar Reategui, Giovanna da Silva, David J. Maron, Steven D. Wexner, Eric G. Weiss

**Affiliations:** Department of Colorectal Surgery, Cleveland Clinic Florida, Weston, FL, USA

**Keywords:** diverticulitis, sigmoidectomy, numbers of prior attacks

## Abstract

**Purpose:** We aimed to investigate the relationship between the number of prior episodes of diverticulitis and outcomes of sigmoid colectomy.

**Methods:** After institutional review board approval, a retrospective review was undertaken based on records of patients who underwent sigmoid resection with anastomosis for diverticulitis between 4 May 2007 and 29 February 2012. Patients were divided into two groups: 0–3 attacks (group 1) and ≥4 attacks (group 2). Statistical analyses were performed to determine whether the groups differed on demographic, intra-operative and postoperative variables.

**Results:** We identified 247 patients who underwent sigmoid colectomy for diverticulitis (45 open, 202 laparoscopic). The two groups did not differ significantly in age, gender, American Society of Anesthesiologists score, past surgical history, body mass index, length of stay, use of a stoma or number of prior hospitalizations for diverticulitis. Group 1 had a higher rate of abscesses (30.6 vs 6.8%, *P* < 0.001) and fistulas (19.4 vs 0.9%, *P* < 0.001); a longer operative time (190.1 vs 166.3 min, *P* = 0.0024); and higher rates of postoperative complications (45.8 vs 23.3%, *P* < 0.001) and conversion (17.1 vs 4.4%, *P* = 0.0091). The most common surgical complications in groups 1 and 2 were wound infection (35 vs 10) and ileus (20 vs 8). Based on multivariate regression analysis, ≥4 attacks were independently correlated with a lower complication rate (odds ratio = 0.512, 95% confidence interval = 0.266–0.987, *P* = 0.046).

**Conclusions:** Patients who had ≥4 previous attacks of diverticulitis had fewer postoperative complications.

## INTRODUCTION

Diverticulitis is a common condition in the western world, the incidence of which is increasing as the population of older adults increases. In industrialized nations, the prevalence of diverticulitis is approximately 5–10% of individuals by 50 years of age. After ages 70 and 85 years, the prevalence increases to 50 and 66%, respectively [1, 2]. Sigmoid colectomy has been the standard, universally accepted surgical treatment for both acute and chronic sigmoid diverticulitis; however, data are lacking to guide predictions of the occurrence or the severity of an attack. Timing for surgery has been widely debated, with some authors reporting that delaying elective sigmoid colectomy for an extended time places the patient at a higher risk of recurrent diverticulitis and therefore increases morbidity and mortality [3, 4].

Some researchers have investigated the relationship between the number of prior diverticulitis episodes and the associated rate of complications; however, a significant correlation has not been established [5]. In addition, studies have been published on the relationship between the number of prior episodes and the conversion rate in laparoscopic surgery for diverticulitis. One study reported a higher conversion rate in patients with three or more diverticulitis episodes requiring hospital admission [6]. In contrast, Natarajan reported no direct relationship between number of episodes and conversion [5].

Currently, many experts agree that the indications for elective surgery include patients with two or more previous episodes of acute diverticulitis who were conservatively treated, and patients with one complicated episode associated with a contained perforation, colonic obstruction, or fistula [7]. According to an earlier iteration of the guidelines of the American Society of Colon and Rectal Surgeons (ASCRS), resection is usually recommended after two uncomplicated attacks of diverticulitis. Conversely, Janes *et al.* found no evidence to support this recommendation [8]. The most recently published ASCRS guidelines state that decisions about whether patients should undergo colectomy after recovery from acute diverticulitis should be made on a case-by-case basis [9].

The aim of this study was to investigate the relationship between the number of prior episodes of diverticulitis and surgical outcomes.

## PATIENTS AND METHODS

After institutional review board approval, a retrospective review of a prospectively maintained database was undertaken, based on the records of patients who underwent sigmoid resection with primary anastomosis for diverticulitis between 4 May 2007 and 29 February 2012. All operations were performed by one of five board-certified colorectal surgeons. Diverticulitis was diagnosed by history, physical examination, blood tests and CT scan. Variables for data collection included age, gender, body mass index (BMI), American Society of Anesthesiology (ASA) score, previous abdominal surgery, pre-operative abscess and fistula, number of prior episodes of diverticulitis, type of surgery (open; laparoscopic), conversion to laparotomy, length of operation, intra-operative and postoperative complications, length of hospital stay and comorbidities. Patients who underwent either Hartmann’s creation or closure were excluded. The definitions of attack included those which (i) required admission, (ii) were clinically diagnosed, (iii) were diagnosed by CT, US or MRI or (iv) were based on the patient’s history. Some of the patients with colovesical, colovaginal or colocutaneous fistula had no recollection of having experienced acute diverticulitis.

Postoperative complications were categorized as surgical complications including bleeding, abscess, anastomotic leakage and wound infection or as medical complications such as pneumonia, thrombosis of the portal vein, congestive heart failure or myocardial infarction, or acute renal failure. Intra-abdominal abscesses were diagnosed through ultrasound or CT scan and were managed by re-operation, antibiotic therapy, CT-guided drainage or a combination of these approaches. The standard definition of surgical site infection (SSI), as described by Mangram *et al.* [10], was used.

The Student’s *t*-test and chi-squared test were used for analyzing continuous and categorical data, respectively. A *P*-value of <0.05 was considered to indicate statistical significance. All univariate predictors with *P* ≤ 0.2 in logistic regression analysis were included in a multivariate analysis to determine the incidence of surgical complications. Receiver operating characteristic (ROC) curve analysis was applied to determine the cut-off value for previous number of attack of diverticulitis.

## RESULTS

The study included 247 patients of a mean age of 55.9 (range 21–83) years who underwent sigmoid colectomy for diverticulitis between 04 May 2007 and 29 February 2012. Based on ROC curve analysis, three attacks was identified as the cut-off value most related to the onset of complications. Patients were divided into two groups: 0–3 attacks (group 1, *n* = 144) and ≥ 4 attacks (group 2, *n* = 103). The two study groups did not differ significantly in age, gender, ASA score, past surgical history, or BMI (Table 1).

**Table 1.** Patient characteristics by study group

Variable	Group1 (≤3 attacks) *n* = 144	Group 2 (≥4 attacks) *n* = 103	*P*
Age (years)	55.6 ± 12.9	56.5 ± 11.0	0.57
Gender (M: F)	72: 72	52: 51	0.85
BMI (kg/m^2^)	28.5 ± 5.5	27.7 ± 5.2	0.23
ASA (1–2: 3–4)	119: 25	91: 12	0.13
Previous abdominal surgery (*n*)	46 (31.9%)	32 (31.1%)	0.80
Comorbidity (*n*)	70 (48.6%)	56 (54.4%)	0.37

Patients in group 1 had a significantly lower mean number of pre-operative hospital admissions (1.1 ± 0.9 vs 1.8 ± 1.9, *P* = 0.0016). Group 1 had significantly higher rates of pre-operative abscesses (30.6 vs 6.8%, *P* < 0.001), fistulas (19.4 vs 0.9%, *P* < 0.001; Table 2) and IR drainage (13.2 vs 0%, *P* = 0.0003). Analyses of intra-operative variables indicated that group 1 had a longer mean operative time (190.1 vs 166.3 min, *P* = 0.0024), greater blood loss (164 vs 117 ml. *P* = 0**.**0004) and higher rates of conversion (17.1 vs 4.4%, *P* = 0.0091), diverting ileostomy (25 vs 3, *P* = 0.0009) and use of ureteric stents (65 vs 29, *P* = 0.0067; Table 3). Although more patients in group 1 had emergency surgery, the difference was not statistically significant (Table 3).

**Table 2.** Pre-operative variables

Variables	Group 1 (≤3 attacks) *n* = 144	Group 2 (≥4 attacks) *n* = 103	*P*
Number of attacks (*n*)	2.1 ± 0.9	6.3 ± 3.9	<0.01
Number of hospitalizations (*n*)	1.1 ± 0.9	1.8 ± 1.9	0.0016
Abscess (*n*)	44 (30.6%)	7 (6.8%)	<0.01
IR drainage (*n*)	19 (13.2%)	0 (0%)	0.0003
Fistula (*n*)	28 (19.4%)	1 (0.9%)	<0.01
Colovesical (*n*)	11 (7.6%)	1 (0.9%)	0.035
Colovaginal (*n*)	10 (6.9%)	0 (0%)	0.016
Enterocolic (*n*)	6 (4.2%)	0 (0%)	0.093
Colocutaneous (*n*)	2 (1.4%)	0 (0%)	0.631

**Table 3.** Intra-operative methods and complications

Variables	Group 1 (≤3 attacks) *n* = 144	Group 2 (≥4 attacks) *n* = 103	*P*
Elective: Emergency (*n*)	134: 10	102: 1	0.054
Open: Laparoscopic (*n*)	33: 111	12: 91	0.024
Ureteric stents (*n*)	65 (45.1%)	29 (28.2%)	0.007
Intra-operative complications (*n*)	11 (7.6%)	4 (3.9%)	0.343
Blood loss (g)	163.9 ± 136.9	117.2 ± 101.4	0.0004
Conversions (*n*)	19/111 (17.1%)	4/91 (4.4%)	0.0091
Diverting stoma (*n*)	25 (17.4%)	3 (2.9%)	0.0009
Operative time (min)	190.1 ± 62.1	166.3 ± 57.5	0.0024

Patients in group 1 had a significantly longer mean length of hospital stay and higher rates of overall postoperative complications (45.8 vs 23.3%, *P* < 0.001) and wound infections (24.3 vs 9.7%, *P* = 0.0034; Table 4). The most common surgical complications for both groups were wound infections, ileus and leaks.

**Table 4.** Postoperative complications and hospital stay

Variables	Group 1 (≤3 attacks) *n* = 144	Group 2 (≥4 attacks) *n* = 103	*P*
Overall postoperative complications (*n*)	66 (45.8%)	24 (23.3%)	0.0003
Respiratory (*n*)	5 (3.5%)	1 (0.9%)	0.401
High stoma output (*n*)	6 (4.2%)	0 (0%)	0.093
Urinary tract infection (*n*)	2 (1.4%)	2 (1.9%)	0.864
Wound infection (*n*)	35 (24.3%)	10 (9.7%)	0.0034
Ileus (*n*)	20 (13.9%)	8 (7.8%)	0.194
Anastomotic leak (*n*)	7 (4.9%)	2 (1.9%)	0.388
Abdominal collection (*n*)	4 (2.8%)	2 (1.9%)	0.999
Percutaneous radiographic drainage (*n*)	1 (0.7%)	1 (0.9%)	0.631
Length of hospital stay (days)	6.5 ± 4.1	5.3 ± 2.6	0.0042

To identify predictors of surgical outcomes, we compared patients with and without surgical complications. Univariate analysis indicated that patients with postoperative complications had significantly lower ratios of ASA 1–2 to 3–4 scores and elective to emergency surgeries (Table 5). In addition, these patients had significantly higher ratios of open to laparoscopic surgeries and higher rates of previous abdominal surgery and pre-operative abscesses or fistulas. The ratio of group 1 to group 2 patients was significantly higher among those who had postoperative complications. Univariate predictors with *P* ≤ 0.2 were included in a multivariate analysis, to determine the incidence of complications. On multivariate regression analysis, ≥4 attacks were independently correlated with a lower complication rate (odds ratio = 0.512, 95% confidence interval = 0.27–0.99, *P* = 0.046) (Table 6).

**Table 5.** Characteristics, comorbidities and operative approaches in patients grouped by absence or presence of postoperative complications

Variables	No postoperative complication *n* = 157	Postoperative complication *n* = 90	*P*
Age (years)	58.0 ± 11.3	57.8 ± 13.3	0.078
Males: Females (*n*)	78: 79	46: 44	0.829
Group 1: 2 (*n*)	78: 79	66: 24	0.0002
Hospitalizations (*n*)	1.5 ± 1.6	1.2 ± 0.9	0.071
BMI (kg/m^2^)	27.0 ± 4.9	30.3 ± 5.6	0.361
ASA 1–2: 3–4 (*n*)	132: 15	68: 22	<0.01
Comorbidities (*n*)	74 (47.1%)	52 (57.8%)	0.106
Previous abdominal surgery (*n*)	41 (26.1%)	37 (41.1%)	0.016
Pre-operative abscess or fistula (*n*)	35 (22.3%)	35 (38.9%)	0.0058
Pre-operative percutaneous radiographic drainage (*n*)	10 (6.4%)	9 (10.0%)	0.310
Open: Laparoscopic (*n*)	18: 139	27: 63	0.0004
Elective: Emergency (*n*)	155: 2	81: 9	0.0016

**Table 6.** Multivariate regression analysis of postoperative complications

Variables	odds ratio	95% confidence interval	*P*
Group 2	0.512	0.27–0.99	0.046
ASA (III–IV)	1.98	0.86–4.53	0.107
Presence of comorbidity	1.18	0.63–2.20	0.613
Pre-op abscess or fistula	1.61	0.83–3.17	0.159
Category (laparoscopic)	0.53	0.24–1.14	0.105
Age	1.02	0.99–1.04	0.246
Status (emergency)	6.07	0.95–38.79	0.056
Previous surgery	1.79	0.97–3.32	0.061

To determine whether the significant difference in complications between groups 1 and 2 might have been influenced by pre-operative abscesses and/or fistulas, we compared outcomes after excluding patients with these conditions (*n* = 82 in group 1; *n* = 95 in group 2). The results still indicated that the rate of overall postoperative complications was significantly greater in group 1 than in group 2; 39.0 vs 24.2% (*P* = 0.034) (Table 7). In addition, we ran this analysis excluding patients with ASA scores of 3 and 4. Group 1 had significantly higher rate of overall postoperative complication (40.3 vs 21.9%, *P* = 0.0048). When patients who underwent emergency surgery were excluded, a significant difference persisted in overall postoperative complications (42.5% in group 1 vs 22.5% in group 2, *P* = 0.001).

**Table 7.** Postoperative complication rates in patients grouped by number of diverticulitis attacks and inclusion/exclusion of abscess or fistula

	Group 1 (≤3 attacks)	Group 2 (≥4 attacks)	*P*
Includes abscess or fistula (*n*)	66 (45.8%)	24 (23.3%)	0.0003
Excludes abscess or fistula (*n*)	32 (39.0%)	23 (24.2%)	0.0337

## DISCUSSION

Although most cases of acute diverticulitis initially respond to non-operative management, surgical intervention remains the standard treatment. The American College of Gastroenterologists currently recommends sigmoid colectomy for patients with two or more attacks of diverticulitis and for those younger than 40 years after the first attack [3, 11–14]. Earlier guidelines from the ASCRS noted that resection is usually offered to patients after two uncomplicated diverticulitis episodes [15]. However, the most current ASCRS guidelines recommend that surgical decisions should be made on a case-by-case basis [9].

Cole *et al.* reported that sigmoid resection after three or more attacks was associated with poor outcomes [6], whereas Natarajan *et al.* did not find a significant relationship between the numbers of prior episodes and the rate of postsurgical complications [5]. In our study, patients who had ≥4 attacks of diverticulitis had fewer postsurgical complications (Table 4). The fact that group 1 (≤3 attacks) had significantly fewer attacks before the need for surgery speaks in favor of a more aggressive disease in this population of patients. This aggressiveness, which usually manifests during the first few attacks, translates into increased morbidity and mortality and has been reported by other authors [16, 17]. Due to the relatively benign course of the disease after the third attack [16], we can suggest that surgery might be offered to patients after four attacks and that, according to our results, waiting until this point might even be beneficial. Unfortunately, the retrospective nature of this review precluded knowledge of the timing of- and interval between the prior attacks.

Some authors have suggested that the indications for surgical treatment of diverticulitis should include complications such as abscess, fistulization, or perforation [7, 18]. In our study, ≤3 attacks was associated with a significantly higher rate of pre-operative abscesses and fistulas. In a study on 150 patients, Chapman *et al.* also found that those with ≤2 attacks had a higher fistula rate (21 vs 9%), although the difference was not significant [19]. Cole *et al.* reported that, although a history of abscess increases the rate for conversion, it does not result in a longer operative time or increased length of stay and complication rates [6]. Our study showed significantly higher rates of abscess in group 1 (30.6 vs 6.8%). Similar results were reported by Chapman in patients with ≤2 attacks, although this difference was not significant [19]. Again, this finding speaks in favor of a more aggressive disease at presentation in some patients; however, the reason for this condition remains unknown. Although, in our study, patients with higher ASA scores had higher rates of complications, ASA did not prove to be a predictive factor for postoperative complications. This finding correlates with a report by Elliot *et al.*, who found that shock on admission, major comorbidity and higher ASA grade were predictive factors of death after surgery for acute complications of diverticular disease [3]. Furthermore, our results show that due to the nature of the diverticulitis in group 1, the preference for using ureteral stents and performing diverting stomas was significantly greater in this group than for patients in group 2. A higher rate of diverting stomas in patients with ≤2 attacks was also reported by Chapman [19]. Surgeries in this group were more challenging and were therefore associated with a significantly greater mean blood loss and a longer operative time and hospital stay, along with a higher rate of conversions, which translated into a higher rate of SSI.

Several investigators have established the feasibility of laparoscopic resection for patients with a history of complicated diverticulitis [20–24]. In a large study with 526 patients who underwent laparoscopic sigmoid resection for recurrent diverticulitis, male gender, anemia, previous myocardial infarction, heart failure and level of surgeons’ experience were independent predictive risk factors for postoperative complications such as anastomotic leakage, SSI and bleeding [25]. The same study reported age (≥75 years) as the only independent risk factor for intra-operative complications like bowel and ureteric injury and bleeding; however, these authors did not report on pre-operative abscess or fistula.

In our study, even after excluding patients with pre-operative abscess or fistula or ASA 3 and 4 scores, the results still indicate that fewer attacks were associated with a greater rate of postoperative complications. Furthermore, on multivariate regression analysis, ≥4 attacks were independently correlated with a lower complication rate. These findings must be considered in the context of our study’s limitations, which are related to its retrospective design and implementation in a single institution. One specific problem is the lack of clarity of the timing and interval between the attacks. A second potential problem is that the episodes of diverticulitis in group 2 could have been milder than group 1. Again, the retrospective nature of this study precludes quantification of severity of the attacks.

## CONCLUSION

Patients with ≥4 prior episodes have fewer complications following surgical resection. Those with ≤3 attacks had a higher rate of pre-operative abscesses and fistulas. The predictors of poor outcomes after surgery for diverticulitis are unclear; further studies are required to investigate this relationship.

**Conflict of interest:** none declared.
